# Polyphenol-rich fermented hempseed ethanol extracts improve obesity, oxidative stress, and neural health in high-glucose diet-induced *Caenorhabditis elegans*

**DOI:** 10.1016/j.fochx.2024.101233

**Published:** 2024-02-20

**Authors:** Simon Okomo Aloo, Kaliyan Barathikannan, Deog-Hwan Oh

**Affiliations:** a Department of Food Science and Biotechnology, College of Agriculture and Life Sciences, Kangwon National University, Chuncheon, Gangwon-do 24341, Republic of Korea; b Faculty of Agriculture and Food Security, Tom Mboya University, Homabay 199-40300, Kenya; c Agricultural and Life Science Research Institute, Kangwon National University, Chuncheon 24341, Republic of Korea; d Saveetha School of Engineering, Saveetha (SIMATS) University, Sriperumbudur, Chennai 600124, India

**Keywords:** Fermentation, *Pediococcus acidilactici SRCM201591*, Neural health, Phenolic identification, Toxicity, UHPLC-Q-TOF-MS/MS^2^

## Abstract

Whole hempseed (WHS), fermented whole hempseed (FWHS), dehulled hempseed (DHS), and fermented dehulled hempseed (FDHS) ethanol extracts were tested for their toxicity and physiological benefits in relation to their phenolic profiles. The safety of all samples was confirmed by the absence of toxic effects on HepG2 cells. FWHS exhibited the highest capacity to inhibit lipase activity (70.80%) and acetylcholinesterase (AChE) (78.94%) in vitro. Similarly, in HepG2 cells, FWHS revealed the greatest ability to reduce the accumulation of reactive oxygen species (ROS). Fermented hempseed demonstrated superior antioxidant, neuroprotective and anti-fat potential, counteracting ageing in high glucose diet-induced *C. elegans* than unfermented. HPLC and UHPLC-Q-TOF-MS/MS^2^ phenolic identification revealed the presence of diverse flavonoids, phenolic acids, lignanamides, and phenylamides in hempseed extracts. Among these polyphenols, quercetin, gallic acid, and kaempferol exhibited excellent antioxidant potential, whereas *N-trans*-feruloyl tyramine displayed the highest anti-lipase potential. This study suggests that polyphenol-rich hempseed exhibits potent antioxidant, and anti-obesity effects, and could improve neural health.

## Highlights

• Fermented hempseeds demonstrated superior anti-obesity and antioxidant capabilities.
• The inhibition capacity of AChE was higher in fermented hempseeds.
• Phenolic compounds are essential components in hempseeds, exerting health benefits.
• Hempseed could provide natural remedies for obesity, oxidative stress, and neurodegeneration.

## 1 Introduction

The incidence of obesity has dramatically increased over the recent past. Obesity is associated with the development of many other disorders, including hypertension, diabetes, heart-related diseases, respiratory disorders, and some types of cancers. It is also reported as one of the major causes of depression and poor life quality among children and middle-aged populations worldwide (Mazon, de Mello, Ferreira, & Rezin, 2017). On the other hand, neurodegeneration is a common cause of cognitive impairment, mainly among older adults. Parkinson's and Alzheimer's are the most common neurodegenerative disorders, affecting millions globally. Although the etiology of neurodegeneration is not well understood, studies have claimed that the disorder is associated with multiple damages in the central nervous system, originating from destructions to the brain cholinergic system, primarily due to the loss of neurotransmitters (mainly, acetylcholine) (Walczak-Nowicka & Herbet, 2021). Among the causative agents, oxidative stress is primarily implicated in neurodegeneration. This disorder can disrupt the typical cellular signalling and defence processes, leading to imbalances that initiate cellular damage in the brain. This phenomenon can be triggered by a range of factors, some of which can be traced to the development of metabolic disorders, such as obesity (Melo et al., 2011). Thus, most recent evidence reinforces the link between obesity, oxidative stress, and neurodegeneration (Melo et al., 2011; Pugazhenthi, Qin, & Reddy, 2017; Rungratanawanich, Qu, Wang, Essa, & Song, 2021). While the molecular aspects underlying this connection are yet to be fully established, the above-referenced studies have speculated that obesity, through increased levels of circulating free fatty acids, can trigger impairment in normal insulin functions, inducing insulin resistance. Insulin resistance incidence progressively leads to oxidative stress by triggering the overproduction of reactive oxygen species (ROS), with superoxide ion (O^-2^), hydrogen peroxide (H_2_O_2_), and hydroxyl radical (OH·) being the major culprits (Melo et al., 2011; Pugazhenthi et al., 2017). Eventually, excessive ROS production initiates neuroinflammation and degradation, which is the ultimate consequence of cognitive decline. Therefore, the combinations of anti-obesity, antioxidants, and neuroprotective agents offer a shield from chronic conditions, which may lead to the destruction of primary neurotransmitters such as acetylcholine (ACh).

*Cannabis sativa* is a plant with hundreds of bioactive compounds produced via secondary metabolism. Hemp plant components (seeds, stems, and oil) possess health benefits, including anti-inflammation, anti-obesity, anti-neurodegeneration, and antioxidants (Aloo, Kwame, & Oh, 2022; Rea et al., 2021, Vanin et al., 2022). These health benefits of hempseed arise mostly from its bioactive compounds, primarily polyphenols, consisting of cannabinoids, phenolic acids, phenylamides, and lignanamides. Fermentation is one of the earliest and most economical bioprocessing methods that could be applied to diversify bioactive components and improve the health effects of plant-based food products. It involves the growth and metabolic activities of microorganisms, which enhance the bioactive metabolites related to the health properties of foods and other products. Fermentation, especially that involving lactic acid bacteria, has been approved as a practical biotransformation approach, enhancing hempseed's health benefits beyond other bioprocessing methods, such as enzyme hydrolysis (Pontonio et al., 2020). The release of bound compounds through the degradation of lignocellulose bonds has mainly been reported as part of the activity of this microbial group, which improves the diversity and the bioavailability of bioactive compounds in fermented products (Verardo, Gómez-Caravaca, & Tabanelli, 2020). To date, only a few studies have been reported on the health benefits of fermented hempseeds (AAloo et al., 2023, Pontonio et al., 2020). Indeed, when this data was being compiled, no report had appeared in the mainstream journals describing the benefits of fermented hempseed on brain health. Thus, the aims of the current study were: (1) to determine the toxicity of industrial hempseed ethanol extracts before and after fermentation; (2) to evaluate the anti-obesity, antioxidant, and neuroprotective properties of unfermented and fermented hempseed ethanol extracts in high-glucose diet-induced-*Caenorhabditis elegans*; (3) to conduct metabolite profile analysis of whole and dehulled hempseed ethanol extracts to reveal their polyphenol composition after fermentation. We hypothesized that fermented industrial hempseed contains an improved phenolic profile, which could improve their health benefits when compared to unfermented ones.

## 2 Materials and methods

### 2.1 Fermentation process

Fermentation was performed using *Pediococcus acidilactici* (*SRCM201591*). The bacterial culture was freshly prepared by inoculating it into a conventional MRS media and then cultured at 37 °C for 24 hrs. Finely ground powders of whole hempseed (WHS) and dehulled hempseed (DHS) were autoclaved. After cooling, the autoclaved samples were weighed into sterilized distilled water (1:3) and mixed thoroughly. The slurry obtained was inoculated with *Pediococcus acidilactici* (201591) (2 × 10^8^ cfu/mL) and maintained at 37 °C with a shaking speed of 140 rpm. After 48 h, the fermentation process was halted. For the unfermented hempseed, whole and dehulled hempseeds were ground, and the ground samples were used in the subsequent experiments.

### 2.2 Preparation of ethanol extracts

Whole hempseed (WHS), fermented whole hempseed (FWHS), raw dehulled hempseeds (DHS), and fermented dehulled hempseeds (FDHS) were weighed into 70 % ethanol in a 1:20 (weight/volume) ratio. Each sample mixture was extracted following the procedure described in our previous article (Aloo, Ofosu, & Oh, 2021). The final supernatant was concentrated under a vacuum, freeze-dried, and then used for further analysis by reconstituting in 70 % ethanol for in vitro studies or 1 % dimethyl sulfoxide (DMSO) for *in vivo C. elegans* analysis.

### 2.3 Total phenolic content

The total polyphenol content (TPC) of ethanol extracts was determined by mixing the individual extracts with folin ciocalteu reagent and then incubating for 2 hrs before adding 800 µL of 700 mM sodium carbonate. The absorbance was measured by spectrophotometer. The detailed procedure for TPC determination was detailed in our previous study (Aloo et al., 2021). TPC was expressed in milligrams per gram of gallic acid equivalent, dry weight (mg/g GAE, DW).

### 2.4 DPPH and ABTS radical inhibition assays

The radical scavenging capacity of the samples was performed based on DPPH and ABTS assays using extracts at 50–400 µg/mL concentrations. Our previous article (Aloo et al., 2021) describes a step by step procedures for the above assays.

### 2.5 Pancreatic lipase inhibition assay

The pancreatic lipase inhibition assay was done following the protocol we already described in our previous work (Aloo et al., 2021). The details, including the formula for calculating the lipase inhibition, have been described in the given reference. The concentrations of 50–400 µg/mL of the extracts were used for the current analysis.

### 2.6 In vitro acetylcholinesterase (AChE) inhibitory activity

In vitro, acetylcholinesteras (AChE) inhibition was investigated following the methodology outlined by (Patel & Ghane, 2021). A specific amount (100 µL) of plant extract (50–400 µg/mL) was combined with 150 µL of AChE solution (0.04 unit). This mixture was then supplemented with 0.1 M pH 8.0 sodium phosphate buffer to achieve a final reaction volume of 800 μL, and the combination was allowed to incubate for 15 min at room temperature. After the incubation, 600 μL of 0.5 mM DTNB was added, and the reaction was initiated by introducing 200 μL of 0.71 mM acetyl thiocholine iodide. Following a 30-minute incubation period, the absorbance of the reaction was measured at 412 nm using spectra plate reader. Galanthamine was used as a drug control. The inhibitory activity of AChE was determined as a percentage based on control (which contained all the reactants except the extract replaced by buffer).

### 2.7 In vitro cytotoxicity and antioxidant activity on Hep-G2 cells

#### 2.7.1 Culture condition

Hep-G2 cells, liver cancer cells were obtained from the American Type Culture Collection (ATCC), Rockville, MD, USA. The cells were grown in Dulbecco's modified Eagle Medium Advanced (d-MEM Advanced). The media was supplemented using 10 % fetal bovine serum (FBS), 2 g/L sodium bicarbonate, and 1 mM sodium pyruvate while being maintained under 5 % carbon dioxide at a temperature of 37 °C.

#### 2.7.2 Cell viability assay

The viability of HepG2 cells was determined calorimetrically using a 3-[4,5-dimethylthiazol-2-yl]-2,5 diphenyl tetrazolium bromide (MTT) assay as described by (Mosmann, 1983). This method assesses the capacity of mitochondria to aid the conversion of MTT bromide into its non-soluble formazan derivative. The concentration of formazan is often quantified using a spectrophotometer. Briefly, the cells were cultured for 24 h in 96-well plates at 4 × 10^4^ cells/well. After this, they were washed using phosphate-buffered saline (PBS) and then treated with different concentrations of hempseed extracts (50–400 μg/mL). The treated cells were washed again and incubated for 1 h with 500 μg/mL MTT. After the 1 h incubation period, the formazan crystals were dissolved in 200 μL/well DMSO. The absorbance was measured calorimetrically at 570 nm.

#### 2.7.3 Intracellular ROS measurement in HepG2 cells

Intracellular ROS accumulation was detected via oxidation-sensitive fluorescent probe 2,7-dichlorodihydrofluorescein diacetate (DCFDA). The HepG2 cells were seeded at 6 × 10^4^ cells/well density and incubated overnight. They were then pretreated with extracts (50–400 μg/mL) and incubated with 20 µM DCFDA at room temperature for 1 h. After incubation, the fluorescence was measured using spectraMax (Seoul, Korea) at 37 °C, excitation 485 nm.

### 2.8 Worm culture and maintenance

Wild-type, N2 *Caenorhabditis elegans* (*C. elegans*), was purchased from the Caenorhabditis Genetics Center (CGC) (the University of Minnesota, Minneapolis, Minnesota, USA). The worms were grown in the nematode growth media (NGM) plates, supplemented with a live *Escherichia coli* (*E. coli*) OP50 lawn. The NGM plates containing worms were maintained at 20 °C throughout the experiment. For the supplementation using plant extracts, the synchronized worms were grown in NGM containing 140 mM 5-fluorodeoxyuridine (FUDR) and *E. coli* OP50.

### 2.10 Establishing a high-glucose diet (HGD) condition

Worms were supplemented with *E. coli* OP50 mixed with 2 %, w/v D (+)- glucose (Sigma Aldrich, St. Louis, MO, USA) to facilitate fat accumulation and cause oxidative stress. Then, the extracts at 400 µg/mL were used to assess the anti-obesity, antioxidant, anti-neurodegeneration, and lifespan extension effects in *C. elegans.*

### 2.9 Worm lifespan

After synchronization, *C. elegans,* L1 larvae were fed with only *E. coli* OP50 bacteria for 3 days. Adult worms were divided into NGM plates containing 140 mM FUDR and treated with a mixture of 2 % glucose and 400 µg/mL plant extracts or 100 µg/mL orlistat (drug control). For the lifespan assessment, each treatment plate contained approximately 50 worms. The positive control (disease control) group consisted of *C. elegans* supplemented with 2 % glucose mixed with 1 % DMSO instead of the extracts. The worms were monitored while counting daily and classified as either alive or dead until all the positive control groups were dead (on the 15th day of treatment). Worms were scored as either or live when they could not respond to touch by a platinum wire.

### 2.10 In vivo acetylcholinesterase (AChE) enzyme activity in *C.* e*legans*

The AChE activity was determined in L4-stage nematodes using a colorimetric method. After treatment exposure for 7 days, approximately 500 nematodes were washed three times with M9 buffer solution and transferred to microcentrifuge tubes. Samples were frozen and thawed 3 times in liquid nitrogen, followed by sonication. After sonication, worms were centrifuged for 10 min at 15,000 rpm, and the supernatants were collected. An aliquot (150 μL) of the supernatant was mixed with a solution containing 0.25 mM 5,5′-dithiobis-2-nitrobenzoic acid (DTNB) and 156 mM acetylthiocholine iodide (ASChI), and subsequently incubated at 30 °C for 5 min. The absorbance was measured using a plate reader (Molecular Devices Korea, LLC, Seoul, Korea) at 405 nm. AChE activity was expressed as a percentage of positive control.

### 2.10 Measurement of intracellular ROS in *C. elegans*

To measure intracellular ROS accumulation, synchronized worms were placed in separate NGM/FUDR until they reached L4. The worms were grouped and supplemented with *E. coli* OP50 and 2 % glucose as a HGD to induce obesity and cause oxidative stress. After 3 days, all the plates were treated with the hempseed extracts/Trolox (400 µg/mL) except the positive control group, where 1 % DMSO was used instead of the extracts. The treatment continued for 7 days. At the end of the period, the worms were washed with M9 buffer and centrifuged. For qualitative ROS analysis, approximately 30 worms were incubated with 50 μM of 2ʹ,7ʹ-Dichlorofluorescin Diacetate (DCFDA) in the dark at 20 °C for 1 h. Following incubation, 10 mM sodium azide solution was added to the worms to paralyze them and the worms were positioned on a glass slide for microscopic examination. Ten (10) randomly selected worms in each group were photographed using a BIOREVO BZ-9000 fluorescence microscope (Keyence Deutschland GmbH in Neu-Isenburg, Germany). The fluorescence of the worms was measured using ImageJ software (National Institutes of Health, Bethesda, MD) and the results were expressed as the mean of the fluorescence intensity, which is related to the ROS content.

The ROS level was quantitatively measured using DCFDA and expressed as a percentage positive control. Briefly, at the end of the specified 7-day treatment, the worms were collected into 100 μL PBS, containing 1 % Tween-20 (PBST). The worms were subsequently subjected to sonication using a Branson Sonifier 250, followed by pipetting them into the wells of 96-well plates that contained DCF-DA at a final concentration of 50 μM in PBS. The sample fluorescence was read using a pectraMax i3 plate reader (Molecular Devices Korea, LLC, Seoul, Korea) at 37 °C with excitation set at 485 nm and emission at 530 nm.

### 2.11 Worm size

The Olympus SZ 61 zoom stereomicroscope and HK3.1 CMOS camera were employed for capturing the images of individual worms. The size of *C. elegans* along its central axis was quantified using ToupViewTM 3.7 software.

### 2.12 Fat deposition assay

In the fat deposition tests in *C. elegans*, we used oil-red and Nile-red staining assays. Briefly, 0.5 g oil-red powder was dissolved in 100 mL isopropanol (5 mg/mL) to make a stock solution of oil-red dye. The oil-red working solution was freshly made by mixing the stock solution with distilled water (3:2 ratio). On the other hand, to make a stock solution of Nile Red dye, 0.5 mg/mL Nile-red stock was weighed into 40 % isopropanol, while the working solution was prepared from the stock. After synchronization, L1 larvae *C. elegans* were deposited into NGM plates containing 140 mM FUDR to inhibit progeny production and allowed to grow for three days. The worms on the 3rd day were treated with OP50 mixed with 2 % glucose and 400 µg/mL extracts or orlistat for 7 days. Approximately thirty (30) adult worms were collected and separately stained with oil-red or Nile-red dyes. The worms were transferred into a 2 % sodium azide (NaN_3_), incubated and then washed using PBS saline to remove excess dye. For fat deposition assay using Nile-red, the worms were transferred to a confocal dish (Cat. No: 102350, SPL Life Sciences, South Korea), where they were observed under an inverted fluorescence microscope with a DP74 camera (Olympus CKX53, Tokyo, Japan). Approximately 10 worms were photographed from each treatment group. For fat deposition using oil-red, stained worms were viewed under a light microscope where 10 worms in each group were photographed. ImageJ software quantified the oil-red and Nile-red intensities (http://imagej.net/).

### 2.13 Triglyceride quantification assay

The triglyceride level was measured using the assay kit (BIOMAX, Seoul, Republic of Korea). After synchronization, *C. elegans* were supplied with *E. coli* bacteria mixed with 2 % glucose and hemp extract or orlistat (200 µg/mL) on the NGM/FUDR plate. The plates were maintained at 20 °C, and on the 3rd day, the worms were supplemented with 2 % glucose and plant extract or orlistat for seven days. After this, 1000 worms were collected using M9 buffer and washed with a mixture of the same buffer and 5 % NP-40. The worms were homogenized for 35 min. The triglyceride assays were carried out following the kit manufacturer's instructions.

### 2.14 QPCR analyses

TRIzol® (Thermo Fisher Scientific, Inc., Middletown, VA) was used to extract total RNA from worm samples. The TRIzol reagent procedure was followed carefully throughout the extraction process. A high-capacity cDNA reverse transcription kit obtained from Thermo Fisher Scientific, Inc., located in Middletown, VA, was utilized, and the process was carried out using a standard thermal cycler from Bio-Rad Laboratories Inc. in Hercules, CA, following the instructions provided by each respective manufacture. The RNA purity was verified under a 260/280 absorbance ratio. The specific primers for the target genes were reported in Supplementary Table 1. qPCR assays were performed using StepOne™ real-time PCR System (AppliedBiosystems, Foster City, CA, USA). In each reaction, a mixture was made by mixing 10 μL of MeltDoctor™ HRM MasterMix, 2 μL of genomic DNA, 0.5 μL of each primer, and 7 μL of double-distilled water. The relative expression levels of the measured genes were calculated using the 2^-△△CT^ method and normalized to *act-1*.

### 2.15 HPLC quantification of phenolic and cannabinoid compounds

An agilent series 1100 HPLC instrument (Agilent, France) equipped with a quaternary pump, a diode array detector and an autosampler was used for analyses. Sample extracts (5 mg/mL) were separated on a C18 column. The mobile phase was a binary solvent system composed of formic acid in water (0.5/99.5 v/v) labelled solution A and formic acid in acetonitrile (0.5/99.5 v/v) named solution B. The linear solvent started from 95 % A − 5 % B, up to 60 % A – 40 % B within 60 min, at 0.8 mL∙min-1. 325 and 280 nm were used for this analysis. The levels of polyphenols were expressed in micrograms/milligrams (µg/mg).

### 2.16 UHPLC-Q-TOF-MS/MS^2^ phenolic identification

The freeze-dried sample was dissolved in 70 % ethanol and subjected to UHPLC-Q-TOF-MS/MS2 analysis. The UHPLC-Q-TOF-MS/MS2 conditions remained consistent with our previous report (Aloo, Park, & Oh, 2023). The spectral data acquisition was performed using MassLynx V4.1 (Waters Corp.). The compounds were identified by referencing the in-house phytochemical library (UNIFI 1.8; Waters Corp.) and confirmed by relevant literature reports.

### 2.17 Statistical analysis

The analyses of statistics were done using GraphPad Prism 8.0 (GraphPad Software, San Diego, USA) ANOVA and Tukey's test at a *p < 0.05* significance level. All the experiments were done in triplicate. The principal component analysis (PCA) was done using OriginPro 2021, while heat map clustering was achieved using ClustVis (https://biit.cs.ut.ee/clustvis).

## 3 Results and discussion

### 3.1 Cytotoxicity on HepG2 cell line

**Fig. 1:**
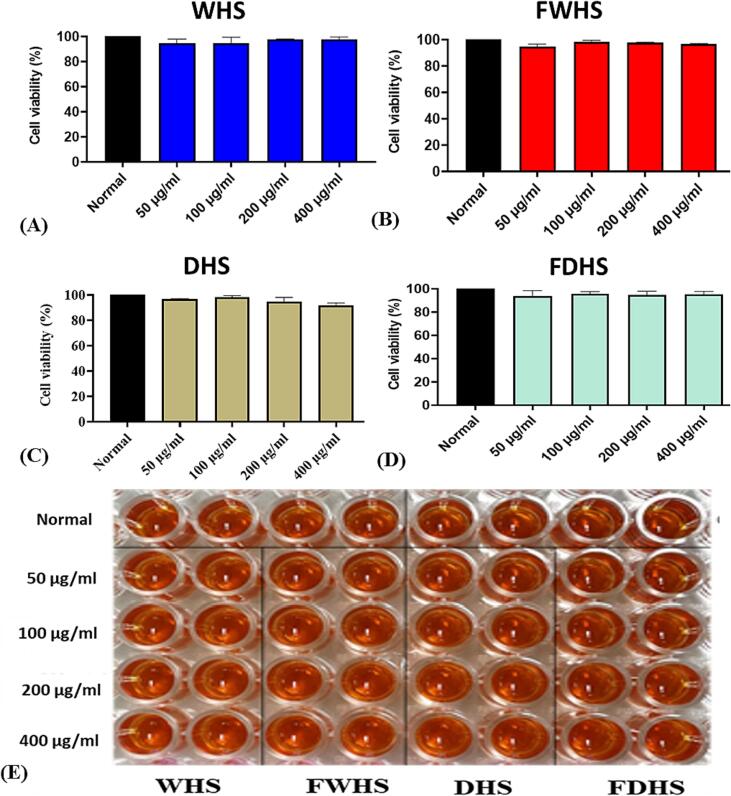
Evaluation of in vitro cytotoxicity of hempseed extracts in HepG2 cells. WHS (whole hempseed), FWHS (fermented whole hempseed), DHS (dehulled hempseed), and FDHS (fermented dehulled hempseed). The corresponding images of the cells subjected to fluorescence assay are depicted in Figure E.

The primary purpose of the in vitro cytotoxicity test is to screen and identify substances with potentially harmful effects that could interfere with crucial cellular functions when the material is consumed. Hence, to evaluate the potential harmful effects of the hempseed extracts, different concentrations (ranging from 50 to 400 µg/mL) were applied to the hepatocellular carcinoma cells (HepG2). The results of the treatment are depicted in Fig. 1A, B, C, and D. Fig. 1E displays the images of the treated cells subjected to the fluorescence assay. Notably, none of the tested extracts demonstrated cytotoxic effects. Even when the concentrations of the extracts were increased to 400 µg/mL, there was no significant impact on cell viability compared to the control group. Our observation contradicts previous findings from cytotoxicity assessments conducted on U-87 glioblastoma cells (Nigro et al., 2020). The reports by (Nigro et al., 2020) revealed that subjecting the U-87 glioblastoma cells to a hempseed lignanamides-rich fraction at seven different doses (0.5, 1.25, 2.5, 5, 10, 25, and 50 µg/mL) resulted in toxic effects after 24 h, 48 h, and 72 h of exposure period. U-87 cell viability was strongly compromised, particularly at the highest concentration tested, at each exposure time assessed (Nigro et al., 2020). Possibly, the chemical composition of hempseed fractions could play a significant role, contributing to the variation between the results we observed and those reported in the literature. Though our work proved to be insightful, future studies should be undertaken to get a clearer picture of the toxicity of hempseed.

### 3.2 Antioxidant capacity

#### 3.2.1 In vitro antioxidant using DPPH and ABTS

Two different antioxidant assessments, namely the ABTS and DPPH scavenging potentials, were employed to measure the in vitro antioxidant efficacy. The antioxidant tests were conducted using the extracts at concentrations of 50–400 µg/mL. At the highest concentration of 400 µg/mL, FWHS exhibited the most robust scavenging activities against DPPH and ABTS, while DHS demonstrated the least potential in vitro antioxidant assays (refer to Supplementary Table 2). The antioxidant capability exhibited a dose-dependent relationship, with 400 µg/mL showing the highest antioxidant efficacy in all assays. Accordingly, investigation using DPPH and ABTS assays illustrated that the fermentation process enhanced the ability of both whole and dehulled hempseeds to prevent free radicals, potentially attributable to elevated polyphenol levels as will be discussed later in the article.

#### 3.2.2 Antioxidant in HepG2 cell line and *Caenorhabditis elegans*

**Fig. 2:**
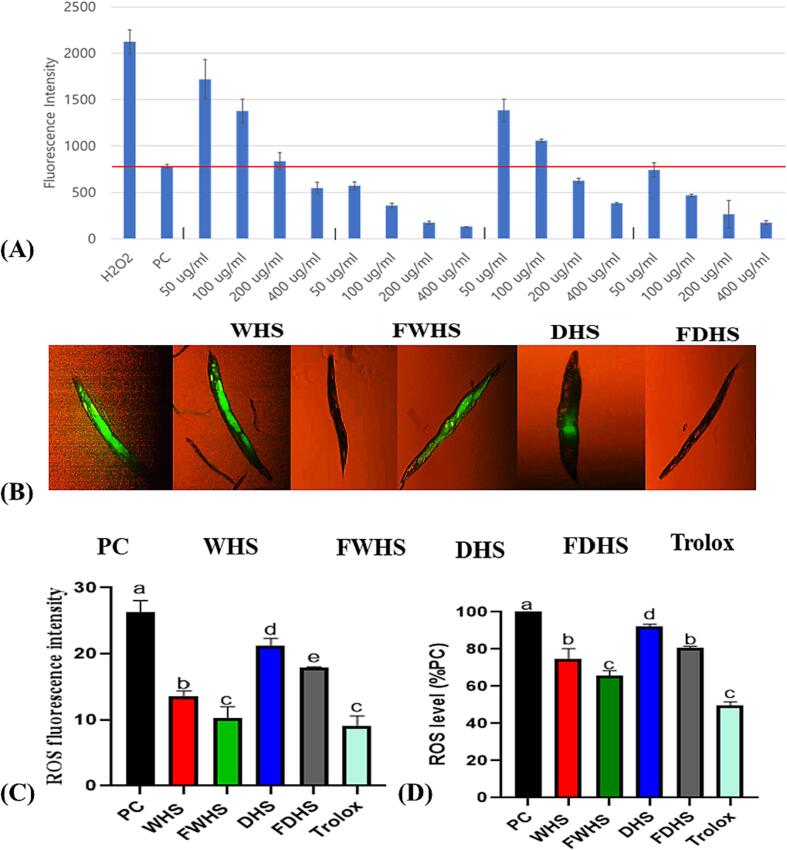
The antioxidant capacity of hempseed extracts as evaluated in HepG2 cells and *C. elegans*. (A) ROS levels in HepG2 cells, (B) fluorescent images depicting ROS accumulation in *C. elegans*, (C) fluorescence intensity of H2D-CFDA-stained *C. elegans*, and (D) ROS levels quantified in *C. elegans*. WHS (whole hempseed), FWHS (fermented whole hempseed), DHS (dehulled hempseed), and FDHS (fermented dehulled hempseed).

Since in vitro assays are only considered as predictive indicators of the potential biological activities, testing extracts directly on human cells or in animals mimicking humans provides more reliable information about their health benefits. Thus, to further assess the antioxidant properties of hempseed extracts, we conducted experiments on human cell model using a hydrogen peroxide (H_2_O_2_)-treated HepG2 cell line. Following HepG2 cell culture, the cells were treated with the extracts for 24 h and then exposed to hydrogen peroxide (H_2_O_2_) as a hepatotoxic substance to induce the accumulation of reactive oxygen species (ROS). Subsequently, the cells were treated with DCF-DA reagent to quantify ROS levels through fluorescence intensity. Notably, fermented whole hempseed (FWHS) emerged as the most effective in reducing ROS accumulation, followed by fermented dehulled hempseed (FDHS), as demonstrated by a concentration-dependent decrease from 50 to 400 µg/mL in Fig. 2A, relative to the positive control (PC). To further predict the actual antioxidant potential of hempseed extracts, we examined the impact of 400 µg/mL extracts on ROS levels in a high glucose-induced *C. elegans* model. Shen and co-researchers previously employed *C. elegans* as a model to investigate the impact of *Citrus aurantium* L. var. amara on reactive oxygen species (ROS) accumulation (Shen, Wan, Wang, & Jiang, 2019). The study highlighted *C. elegans* as an effective model for understanding how plant extracts influence oxidative stress levels. In the current study, the microscopic images of H_2_D-CFDA-stained nematodes were presented in Fig. 2B. The addition of 2 % glucose significantly elevated ROS levels in the PC group compared to the treated groups. Thus, the most significant ROS accumulation occurred in the PC group, followed by the DHS-treated group. In contrast, the FWHS-treated group exhibited the least accumulation, as confirmed by fluorescent intensity (Fig. 2C). Consistent with the microscopic analysis, quantification of ROS concentration indicated reduced levels in all hempseed extract-treated worms compared to the PC group. Dose-response analysis revealed that FWHS extract-treated worms displayed the most substantial reduction in ROS accumulation, indicating its efficacy in inhibiting high-glucose-diet-induced oxidative stress in the worms (Fig. 2D). However, at the same concentration, WHS and DHS exhibited comparatively lower effects on ROS levels. Specifically, FWHS reduced ROS levels by 32 %, while FDHS attained a 42 % reduction. The improved antioxidant potential of fermented samples could be due to the enhanced profile of antioxidant compounds in hempseed, primarily polyphenols after fermentation. Pontonio et al. showed that biotransformation techniques such as fermentation could diversify polyphenol profiles, leading to hempseed products with improved antioxidant capacities (Pontonio et al., 2020). Pontonio and colleagues discovered that lactic acid fermentation, in comparison to other bioconversion methods such as enzyme hydrolysis, was the most effective in enhancing hempseed's antioxidant potential by facilitating diversification and bioavailability of polyphenols (Pontonio et al., 2020). They reported that an enhanced phenolic profile reportedly improved the protective effect of hempseed against oxidative stress in the human keratinocyte cell line (Pontonio et al., 2020).

### 3.3 Anti-obesity, anti-neurodegeneration, and anti-ageing capacities

#### 3.3.1 In vitro enzyme (pancreatic lipase and acetylcholinesterase) inhibitions

**Fig. 3:**
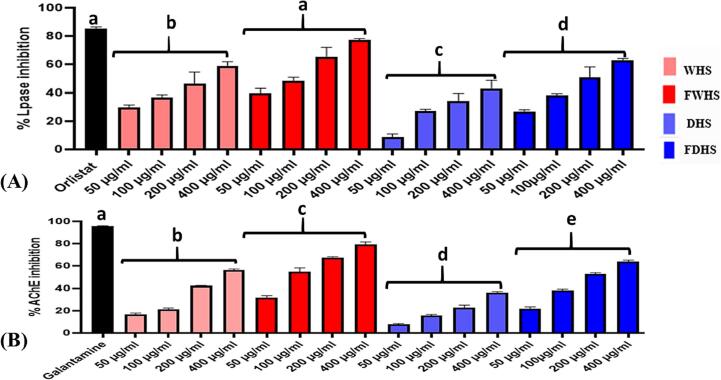
Concentration-dependent (50–400 µg/mL) effects of hempseed ethanol extracts on in vitro pancreatic lipase (A) and AChE (B) activities. WHS (whole hempseed), FWHS (fermented whole hempseed), DHS (dehulled hempseed), and FDHS (fermented dehulled hempseed). The letters indicating statistical differences were generated using the inhibition rate of the extracts at a concentration of 400 µg/mL.

One of the goals of this study was to assess how fermentation affects the anti-obesity potential of hempseed. Pancreatic lipase, a crucial enzyme in obesity development, plays a key role in hydrolyzing triglycerides into fatty acids and glycerol, facilitating fat absorption. As illustrated in Fig. 3A, hempseed extracts at various concentrations (50–400 µg/mL) exhibited dose-dependent effects against lipase activities. At a 400 µg/mL concentration, WHS, FWHS, DHS, and FDHS inhibited lipase activities by 50.94 %, 70.80 %, 38.80 %, and 53.85 %, respectively. These findings suggest that fermented hempseeds (FWHS and FDHS) demonstrated a more potent potential for inhibiting lipase compared to their non-fermented counterparts (WHS and DHS). Previous literature has highlighted fermentation as an effective bioprocess technology for enhancing the lipase inhibition of plant-based foods (Moon and Kim, 2018, Yan et al., 2022). After 72 h of fermentation using *Lactobacillus fermentum* grx08, the lipase inhibitory activity of shenheling extract significantly increased (Yan et al., 2022). Additional findings from this research indicated a positive correlation between the lipase inhibition rate and the total polyphenols and flavonoids (Yan et al., 2022), suggesting that the increased polyphenol content in fermented foods is related to their anti-obesity effects.

Another aim of the present investigation was to assess the impact of fermentation on the neuroprotective potential of hempseed. Neurodegenerative disorders are primarily characterized by the gradual degradation of neurotransmitters, particularly acetylcholine. Acetylcholinesterase (AChE) is the key enzyme associated with the decline in acetylcholine levels. Consequently, the primary strategy for preventing neurodegeneration by plant extracts involves inhibiting AChE-mediated degradation of acetylcholine (Leonard, Zhang, Ying, Xiong, & Fang, 2021; Yan et al., 2015). The in vitro tests of hemp seed extracts revealed a dose-dependent AChE inhibition activity (Fig. 3B). At a 400 µg/mL dose, FWHS extract demonstrated the most substantial AChE inhibitory capacity at 78.94 %, followed by FDHS at 65.34 %, while DHS exhibited the weakest inhibition capacity, irrespective of concentration. While Leonard et al. previously established the anti-AChE effects of unfermented hempseed (Leonard et al., 2021), our study suggests that fermented whole hempseed may serve as a more potent source of bioactive ingredients against neurodegenerative disorders compared to unfermented ones.

#### 3.3.2 Anti-obesity in *Caenorhabditis elegans*

**Fig. 4:**
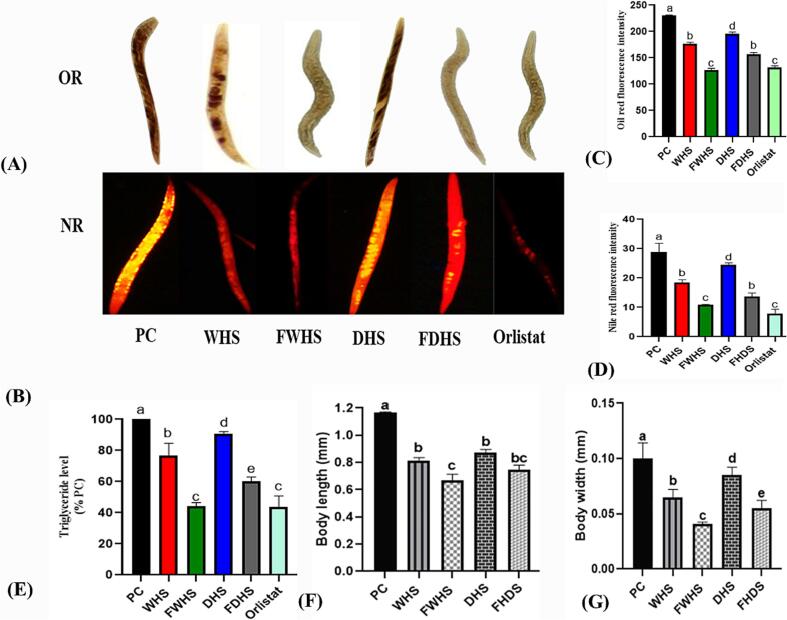
Effects of hempseed extracts on fat accumulation, triglycerides, and body size of high-glucose diet-induced-*Caenorhabditis elegans*. Fat accumulation was measured by Oil-red and Nile-red (A, B), the fluorescence intensity of oil-red and Nile-red-stained worms (C, D), triglyceride levels (E), and body length and width (F, G). OR (oil-red), NR (Nile-red), WHS (whole hempseed), FWHS (fermented whole hempseed), DHS (dehulled hempseed), and FDHS (fermented dehulled hempseed). (For interpretation of the references to colour in this figure legend, the reader is referred to the web version of this article.)

The wild-type Caenorhabditis elegans may serve as an in vivo model to evaluate the influence of plant extracts on obesity-related parameters. We examined the in vivo effect of hempseed ethanol extracts on obesity development using *C. elegans* as a model. First, we assessed the impacts of the extracts on fat deposition (accumulation) in the worms. Fat deposition in Nile-red- and oil-red-stained worms was visualized under a microscope (X10 magnification) and quantified using ImageJ software, measuring fluorescence intensity as pixels per unit area. The intensity of fat deposition was higher in the PC group than in extract-treated groups as demonstrated in Fig. 4 A and B. Thus, all treatments resulted in reduced fat accumulation in the worms compared to the positive control group. Fermented whole hempseed (FWHS) exhibited the most potent anti-fat effects, followed by FDHS, as indicated by decreased fluorescence intensity (Fig. 4 C and D). Additionally, triglyceride levels in the worms were quantified and reported in Fig. 4E. FWHS demonstrated the most potent activity in reducing triglyceride levels, followed by FDHS, consistent with the Nile-red and oil-red assays. More microscopic images of Nile-red and oil-red stained worms have been provided in Supplementary Figs. 1 and 2. Finally, we assessed the effects of the extracts on worm sizes. Fig. 4 F and G illustrate the body length and width measured for the worms, respectively. The worms revealed distinct body measurements when compared to the positive control (PC) group. All the extract-supplemented groups showed relatively smaller sizes. Among these extract-treated groups, the *C. elegans* supplemented with FWHS extract exhibited the most significant reduction in body width (0.04 mm) and body length size (0.70 mm) followed by the FDHS extract-treated group. Generally, all the groups supplemented with fermented hempseed demonstrated a more reduced body size than their counterparts. Similar findings have been reported by (Zhao et al., 2023), suggesting that natural extracts from plant origins could decrease obesity development in *C. elegans*. Fermented hempseed extracts exhibited improved anti-fat effects in *C. elegans* than their non-fermented counterparts.

#### 3.3.3 Anti-neurodegeneration and anti-ageing capacities in *Caenorhabditis elegans*

**Fig. 5:**
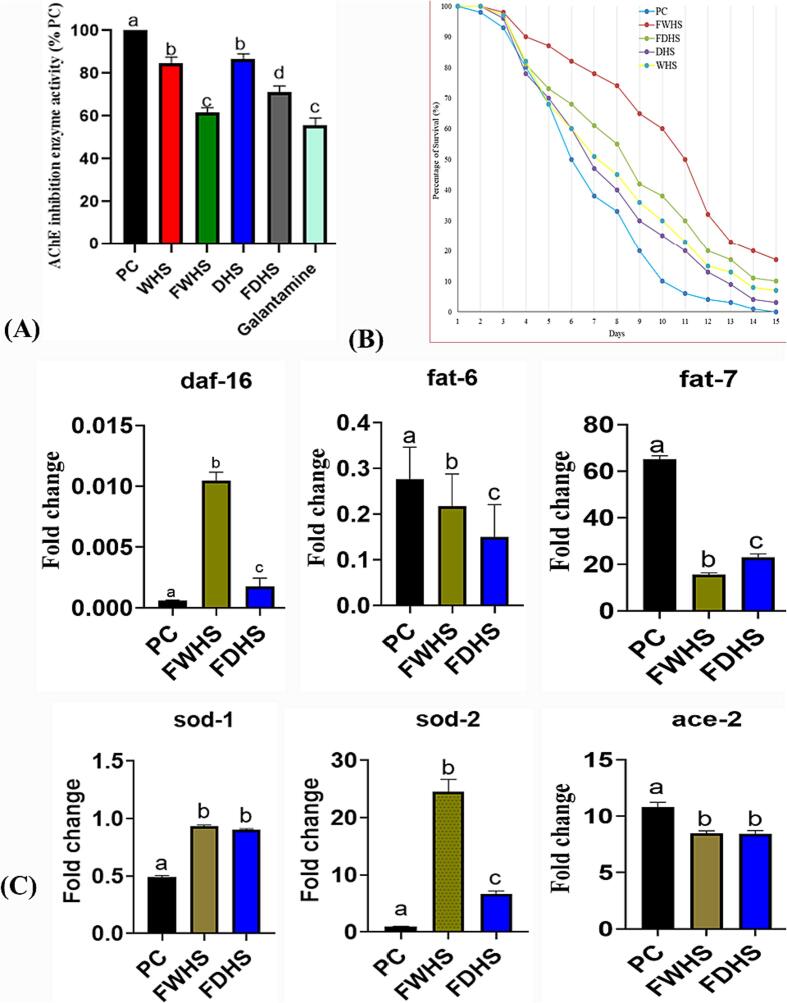
Impacts of hempseed extracts on AChE activity, lifespan, and gene expression in *Caenorhabditis elegans* under glucose supplementation. The effects on AChE inhibition (A), life extension benefits (B), and alterations in gene expression (C). WHS (whole hempseed), FWHS (fermented whole hempseed), DHS (dehulled hempseed), and FDHS (fermented dehulled hempseed).

In previous experiments, researchers have utilized *C. elegans* as a living model to assess the impact of plant extracts on mitigating neurodegeneration and uncovering the associated mechanisms (Wang & Zheng, 2022). Similarly, our present study investigated the neuroprotective potential of fermented hempseed extracts by assessing their inhibitory effects against acetylcholinesterase (AChE) in *C. elegans.* The results (Fig. 5A) demonstrated that the FWHS extract displayed the most potent in vivo inhibitory effect against AChE activities compared to the other tested extracts. In comparison to the PC group, FWHS exhibited a significant 37 % reduction in AChE activity, while WHS, DHS, and FDHS extracts showed inhibitory effects of 18 %, 14 %, and 28 %, respectively. These findings align with our previous in vitro result (Fig. 3B), indicating that fermented hempseed is more effective as an anti-neurodegenerative agent than unfermented hempseed.

Finally, regarding the biological activities of hempseed, we evaluated the anti-ageing effect of the extracts. As illustrated in Fig. 5B, all fermented hempseed extracts demonstrated a more pronounced effect in extending the lifespan of the worms compared to non-fermented hempseed extracts. Among the treatments, FWHS extracts significantly impacted the worms' longevity, displaying the strongest ability to extend the life span, while DHS extract exerted the least effect. Importantly, the maximum lifespan of the model worms (PC group) was 15 days, shorter than all the treated groups. Literature suggests that *C. elegans*’ lifespan is related to their neurological state and susceptibility to metabolic disorders like obesity and oxidative stress (Salmon, 2016). Cogliati and colleagues reported anti-ageing effects of *B. subtilis* in *C. elegans*, attributed to the bacteria's anti-neurodegenerative properties (Cogliati et al., 2020). Similarly, extending *C. elegans'* lifespan through plant extract supplementation was linked to enhanced resistance against oxidative stress induced by lipid accumulation (Zheng et al., 2014). Therefore, we attribute the anti-aging properties of hempseed extracts to their antioxidant, anti-AChE, and anti-fat properties. Consequently, we speculate that fermented hempseed may extend the worms' lifespan by enhancing their ability to counteract the detrimental effects of HGD-induced fat accumulation, ROS buildup, and AChE activity.

### 3.4 QPCR analyses of related genes

To elucidate the underlying mechanisms behind the physiological benefits of fermented hempseed described above, we explored the impact of the extracts on the regulation of relevant genes. In *C. elegans*, genes associated with lifespan extension, fat accumulation, oxidative stress, and acetylcholinesterase activity have been identified. The major transcription factor responsible for regulating various activities in *C. elegans*, including life span, stress resistance, and fat accumulation, is daf-16 (Ogg et al., 1997). Previous research by Ogg and colleagues indicated that daf-16 is down-regulated in metabolic conditions such as diabetes and obesity, contributing to the pathogenesis of these illnesses in *C. elegans* (Ogg et al., 1997). In the current experiment, FWHS and FDHS significantly up-regulated the mRNA expression of daf-16, with the highest fold change observed in the FWHS-treated worms (Fig. 5C). This finding is consistent with the results in Fig. 5B, in which *C. elegans* supplemented with FWHS exhibited the most extended lifespan among the treated groups. On the other hand, *C. elegans* possesses five genes coding for superoxide dismutase (SOD), an enzyme catalyzing the dismutation of the superoxide radical into ordinary molecular oxygen (Back, Matthijssens, Vlaeminck, Braeckman, & Vanfleteren, 2010). The majority of SOD activity in adult *C. elegans* is provided by sod-1 and sod-2 genes (Back et al., 2010). Several recent studies have indicated that up-regulating SOD-related genes is associated with improved resistance to oxidative stress in *C. elegans* (Back et al., 2010, Cabreiro et al., 2011, Lin et al., 2021). In the current study, treatments with fermented hempseed extracts up-regulated the expression of the sod-1 and sod-2 genes. The most significant up-regulation of sod-1 was observed in FWHS-treated groups (Fig. 5C), confirming that the up-regulation of SOD genes is linked to resistance against oxidative stress in *C. elegans.* Moreover, we assessed the impact of fermented hempseed extracts on gene expression related to fat metabolism to unveil their role as anti-obesity agents. The extracts decreased the expression of fat-6 and fat-7 genes involved in fat synthesis, indicating that the anti-fat effects of fermented hempseed extracts in *C. elegans* result from the down-regulation of genes responsible for fat synthesis (Fig. 5C). Lastly, we explored the potential effects of the extracts on AChE-related genes. In vertebrates, only one gene encodes AChE, whereas in nematodes, three genes (ace-1, ace-2, and ace-3) encode this enzyme (Arpagaus et al., 1994). These genes, ace-1, ace-2, and ace-3, represent three distinct catalytic types of acetylcholinesterases (classes A, B, and C), differing in their substrate and inhibitor specificities (Arpagaus et al., 1994). Xin et al. found that compounds derived from Lycoris significantly inhibited the ace-1 and ace-2 genes, thereby preventing AChE activities, modulating neural loss, and reducing alpha–beta (Aβ)-toxicity in *C. elegans* (Xin et al., 2013). In accordance with these findings, Fig. 5C illustrates that the intervention of all fermented hempseed extracts significantly downregulated ace-2, although no observable effect was noted on ace-1 (data not shown). Therefore, hempseed could reduce the degradation of acetylcholine by decreasing the expression of genes aiding AChE activity.

### 3.5 Phenolic heritage of hempseed extracts

Over decade, the significance of polyphenols in hempseeds has been overlooked due to the abundance of proteins and polyunsaturated fatty acids, which have been traditionally perceived as the primary contributors to their high nutritional benefits. Hempseeds contain a diverse array of polyphenols, encompassing flavonoids, phenolic acids, lignanamides, and phenylamides. Although these compounds have the potential to exhibit diverse biological activities, their exploration has been somewhat limited in the past decades due to the reasons described above. Polyphenolic compounds could help improve health by reducing feeding intake, exhibiting bactericidal effects, as well as demonstrating antioxidant and anti-inflammatory activity (Chen et al., 2012, Di Palo et al., 2022). An increase in the total polyphenol content of hempseed samples was observed after the fermentation process in the current study. The highest amount of TPC was observed in FWHS (45.71 ± 0.67 mg/g GAE), while the lowest content was found in DHS (16.58 ± 0.59 mg/g GAE) as shown in Table 1. Recent insights into the polyphenols of hempseed highlight an increase in the phenolic content after fermentation (Pontonio et al., 2020). The present study offers more updated research, underscoring the significance of fermentation in augmenting the total polyphenol content of hempseed, noting an increase in the fermented material (Table 1).

**Table 1:** Total polyphenol content and the list of individual phenolic compounds identified in hempseed ethanol extracts (RT, retention time; N.D, not detected).

Number	Compound	RT	WHS	FWHS	DHS	FDHS
–	TPC (mg/GAE/g)	**–**	21.62 ± 0.10^a^	45.71 ± 0.67^b^	16.58 ± 0.59^c^	23.57 ± 0.29^d^
**HPLC-identified polyphenols (µg/mg)**						
1	Quercetin	42.983	1.065	1.013	N.D	0.7135
2	*N-trans*-feruloyl tyramine	22.370	2.40	2.79	N.D	0.61
3	Apigenin	49.783	0.236	0.822	N.D	N.D
4	Rutin	26.355	0.101	N.D	N.D	N.D
5	*p.* coumaric acid	22.630	0.068	0.114	0.149	N.D
6	Gallic acid	5.667	N.D	N.D	N.D	1.7717
7	Naringin	32.058	N.D	0.042	N.D	N.D
8	Ferulic acid	26.258	0.076	N.D	0.173	0.166
9	Kaempferol	51.336	2.008	3.657	3.149	3.805
10	Genistein	49.459	0.213	0.135	N.D	N.D
11	Catechin	14.680	N.D	N.D	5.3544	4.748
12	Caffeic acid	17.475	N.D	N.D	1.9469	2.303

Next, HPLC and UHPLC-ESI-QTOF-MS^2^ were employed to identify individual phenolic compounds of hempseed extracts. Twelve (12) phenolic compounds were identified through HPLC. Table 1 illustrates the polyphenols that were identified using HPLC in hempseed ethanol extracts. The fermentation process significantly impacted the diversity of these individual polyphenols in hempseed. The process decreased polyphenols such as catechin and ferulic acid in the fermented samples; a decline which could be due to the polymerization of the released phenolic compounds during the later stages of the fermentation process (Dulf, Vodnar, Dulf, & Tosa, 2015). In contrast, the levels of phenolic compounds, such as *N-trans-f*eruloyl tyramine and kaempferol, were increased in fermented hempseeds. This increase could probably be due to the release of bound polyphenols from seed components, a process involving lignin-degrading and carbohydrate-cleaving enzymes produced by the fermenting bacterial culture (Dulf et al., 2015). Scientific evidence has shown that the bonds between polyphenol and lignocellulosic material are weakened during fermentation, facilitating the release of bound compounds and improving their bioavailability (Adebo & Gabriela Medina-Meza, 2020). Consistent with the current findings, the quantification of bioactive compounds showed a significantly changed profile of polyphenols in raw and fermented hempseed (Pontonio et al., 2020). In the study by Pontonio and colleagues*, N-trans*-feryroyltyramine, reportedly increased after fermentation, while relevant changes were also noted in the profile of phenolic acids, resulting from lactic acid bacteria bioconversion pathways of polyphenols.

An untargeted metabolomics approach using UHPLC-ESI-QTOF-MS^2^ made it possible to tentatively identify phenolic compounds whose standards could not be obtained. The UHPLC-ESI-QTOF-MS^2^ chromatograms obtained are shown in Supplementary Fig. 3. Sixteen (16) polyphenols (Table 2) were identified using UHPLC-ESI-QTOF-MS^2^, consisting of phenolic acids, lignanamides, phenylamides, and lignin glycoside. Peak 1 showed a protonated molecular ion discovered at *m*/*z* 147.05, molecular formula of C9H8O2, and it displayed an ion fragment at *m*/*z* 103. This compound was tentatively identified as cinnamic acid supported by appropriate literature evidence (Ciucure, Geana, Sandru, Tita, & Botu, 2022). Peak 2 was identified as feruloyl glucoside, a ferulic acid derivative with reference to literature information (Al-Yousef et al., 2020). Feruloyl glucoside has been characterized in many other plant materials, such as orange juice extract (Oliveras-López et al., 2016), and it is reported to exert antioxidant effects via inhibiting the oxidation of liposomes emulsions (Kylli et al., 2008). Moreover, with the help of an in-house phytochemical library (UNIFI 1.8; Waters Corp.), phloretic acid (peak 3) was characterized as a phenolic acid according to its molecular ion and formula.

**Table 2:** List of UHPLC-ESI-QTOF-MS identified phenolic compounds in hempseed ethanol extract.

Peak no.	RT	Chemical formula	[M-H]-(*m*/*z*)	Fragment pattern	Tentative identification	Peak response areas			
						WHS	FWHS	DHS	FDHS
1.	0.98	C9H8O2	147.05	103	Cinnamic acid	0	0	5955	5376
2.	1.16	C16H20O9	355.10	178, 193	Feruloyl glucoside	26,465	684	0	0
3.	2.33	C18H22O10	397.11	235, 217, 191	Picraquassioside A	16,465	36,465	0	0
4.	2.68	C9H10O3	165.06	119, 147, 165	Phloretic acid	0	90,254	0	106,920
5.	3.21	C17H17NO4	298.11	163,121, 145	*N-trans*-caffeoyl tyramine	489,144	551,895	0	0
6.	3.66	C34H30N2O8	593.19	293, 430, 456	Cannabisin A	201,237	165,887	0	0
7.	3.75	C34H32N2O8	595.21	269, 432	Cannabisin B	275,781	471,263	0	0
8.	3.77	C17H17NO3	282.11	119	*N*-*trans*-coumaroyltyramine	0	89,738	0	0
9.	4.16	C35H34N2O8	609.22	283, 446	Cannabisin C isomer	54,371	81,629	0	0
10.	4.25	C35H34N2O8	609.22	430,336,446	Cannabisin C isomer	63,424	104,478	0	0
11.	4.41	C35H34N2O8	609.22	–	Cannabisin C isomer	0	23,789	0	0
12.	4.67	C36H36N2O8	623.24	267,444, 460	Cannabisin D isomer	23,023	19,096	0	0
13.	4.78	C36H36N2O8	623.24	–	Cannabisin D isomer	18,401	12,688	0	0
14.	5.80	C36H36N2O8	623.24	–	Cannabisin D isomer	139,169	80,271	0	0
15.	5.85	C36H36N2O8	623.24	–	Cannabisin D isomer	88,916	0	0	0
16.	11.12	C22H30O4	357.21	107, 311, 313	Cannabidiolic acid	1,019,393	232,410	0	0

Hempseed extract contained phenylpropanoid amides and their oxidative derivatives, lignanamides in a significant number. These compounds have been reported in previous studies as the main phenolic constituents of most hempseeds (Di Palo et al., 2022, Irakli et al., 2019, Nigro et al., 2020). Peak 3 was tentatively identified with the help of fragment ions at *m*/*z* 235, 217, 191, and 161 as shown in Table 2. The spectral information for peak 3 resembled those of a compound previously isolated from fresh fruits of *Picrasma quassioides*, classified as a phenylpropanoid (Yoshikawa, Sugawara, and Arihara, 1995). Yoshikawa and colleagues identified the compound as picraquassioside A, leading to its characterization in the current study. Peak 5 exhibited a protonated molecular ion at *m*/*z* 298.11. It was identified as phenylamides, representing *N-trans-*caffeoyl tyramine. The MS^2^ analysis of *N-trans*-caffeoyl tyramine revealed a fragment ion at *m*/*z* 163, 121, and 145, resulting from the breakage of the CO-Cα bond of the amide portion as described by (Leonard, Xiong, Zhang, Ying, & Fang, 2021). Regarding the studied biological effects, *N-trans-*caffeoyl tyramine possesses potent inhibitory effects against neurodegeneration, preventing the development of Alzheimer's disease (Di Palo et al., 2022). Peak 6 displayed a protonated molecular ion at *m*/*z* 593.19 and its molecular formula was provided as C34H30N2O8. This compound was confirmed as cannabisin A, a lignanamide. The identification was made possible by its fragment ions at *m*/*z* 293, 430, and 456. Moreover, peak 7, which displayed protonated a molecular ion at *m*/*z* 595.21 and ion fragments at *m*/*z* 269 and 432, was identified as cannabisin B. Together with *N-trans*-caffeoyltyramine, cannabisin B isolated from industrial hempseed reportedly modulated the miRNome of human neural cells with effects on specific microRNAs related to neural functions, exerting anti-alzheimer’s disease potential (Di Palo et al., 2022). Peak 8 showed a protonated molecular ion at *m*/*z* 282.11 and was identified as *N-trans-c*oumaroyltyramine with the help of a fragment ion pattern at *m*/*z* 119. Peak 9, 10, and 11 were primarily cannabisin C isomers, present in both WHS and its fermented form, FWHS. Their tentative identification was facilitated by referring to findings by (Nigro et al., 2020), which allowed a direct comparison of fragment patterns of the compounds. Additionally, peaks 12, 13, 14 and 15 were comprised of cannabisin D isomers. The majority of these phenylamides and lignanamides have been described as the primary metabolites exhibiting health benefits such as anti-neurodegeneration effects in hempseed (Di Palo et al., 2022, Rea et al., 2021). Finally, with the help of its fragment pattern at *m*/*z* 107, 311, and 313, supported by suitable literature reference (Martinez, Montserrat-de la Paz, De la Puerta, García-Giménez, & Fernández-Arche, 2020), peak 16 was tentatively identified as cannabidiolic acid. Findings from scientific research supported cannabidiolic acid as an anti-obesity compound (Ben-Cnaan et al., 2022).

#### 3.5.1 The correlation of UHPLC-quantified polyphenols with in vitro antioxidants and anti-obesity

**Fig. 6:**
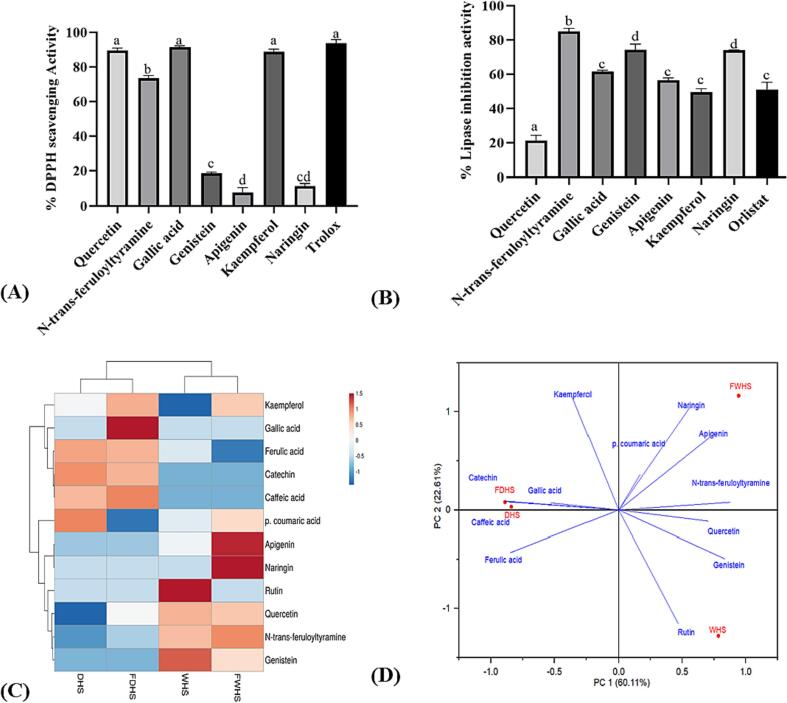
The anti-obesity (A), antioxidant (B), heatmap (C), and PC bi-plots (C) of HPLC-quantified in hempseed. The standards for the HPLC-identified polyphenols were utilized for anti-obesity and antioxidant assessments.

Phenolic compounds present in hempseed have been recognized for their potent antioxidant and anti-obesity effects (Chen et al., 2012, Di Palo et al., 2022). To further elucidate their potential role in the observed benefits of hempseed, we conducted a study to explore the correlations between HPLC-identified polyphenols and the in vitro antioxidant (ABTS and DPPH inhibitions) and pancreatic lipase inhibition activities, employing Pearson's correlation analysis (Refer to Supplementary Table 3). The total polyphenols demonstrated a strong positive correlation with ABTS (r, 0.988), DPPH (r, 0.936), and lipase inhibition (r, 0.959) capacities. This observation aligns with previous literature, indicating a significant (p < 0.01) positive correlation between total polyphenols and the antioxidant activities of hempseed (Irakli et al., 2019), and it highlights the role of total phenolic compounds in contributing to the health benefits of hempseed. However, it was noted that while some of the studied individual polyphenols exhibited a positive correlation with in vitro antioxidant and lipase inhibition capacities, others showed a negative correlation with these activities. For example, caffeic acid, catechin, and rutin predominantly displayed a negative correlation, whereas quercetin, kaempferol, naringin, apigenin, gallic acid, genistein, and *N-trans*-feruloyl tyramine exhibited a positive correlation with both ABTS, DPPH, and lipase inhibitions (Refer to Supplementary Table 3). To further validate their contributions to these health benefits of hempseed, we tested the in vitro DPPH and lipase inhibition potentials of 200 µg/mL of the HPLC-grade quercetin, kaempferol, naringin, apigenin, gallic acid, genistein, and *N-trans*-feruloyl tyramine. The results of this analysis are depicted in Fig. 6A and B.

While no significant differences were observed in the DPPH scavenging capacities of quercetin, gallic acid, and kaempferol, it was evident that naringin and apigenin standards, despite exhibiting the strongest positive correlation with antioxidant capacity, showed limited potential to scavenge DPPH radicals. The recorded DPPH inhibition values were as follows: quercetin (89.44 %), kaempferol (88.77 %), naringin (11.52 %), apigenin (7.78 %), gallic acid (91.48 %), genistein (18.39 %), and *N-trans-*feruloyl tyramine (73.56 %). Additionally, the in vitro lipase inhibitions of these standard compounds were observed in the following order: *N-trans*-feruloyltyramine (84.9 %) > genistein (74.1 %) > naringin (73.8 %) > gallic acid (64.40 %) > apigenin (56.3 %) > kaempferol (49.4 %) > quercetin (21.3 %). Thus, gallic acid (91.48 %) exhibited the most potent antioxidant potential, while *N-trans*-feruloyltyramine (73.56 %) demonstrated the strongest lipase inhibition capacity. It is crucial to note that many of these tested polyphenols have been reported to possess antioxidant and anti-obesity effects. *N-trans*-feruloyltyramine, identified in *Spartina anglica,* was reported to inhibit both ABTS (IC_50_, 8.19 μg/mL) and DPPH (IC_50_, 4.67 μg/mL) but not lipase (Kim et al., 2021). The anti-obesity and antioxidant potential of genistein, naringin, gallic acid, apigenin, kaempferol, and quercetin have been reported before (Hsu and Yen, 2007, Nisha et al., 2014). In summary, the current findings emphasize that the polyphenol heritage of hempseed contributes significantly to their health benefits. Nevertheless, this research exclusively examined the phenolic composition of ethanol extracts of hempseeds. Consequently, the investigation only focused on a limited proportion of the whole polyphenols and bioactive compounds found in hempseeds to make a conclusion on their health benefits. Future research should explore the health-promoting properties linked to non-ethanol extracts, encompassing bioactive compounds extractable through alternative solvents.

#### 3.5.2 Heatmap visualization principal component analysis (PCA) of HPLC-quantified polyphenols in hempseed extracts

Metabolomics data can be visualized by a heatmap that shows the relative amount of each compound in a sample through the intensity of the colour. In the current study, the heatmap was constructed to display how HPLC-identified polyphenols were distributed among the hempseed samples. When subjected to heatmap analysis, the four hempseed extracts showed distinct clusters as demonstrated in Fig. 6 C. Specifically, DHS and FDHS formed one cluster, while WHS and FWHS constituted another cluster. Notably, FWHS showed the highest levels of polyphenols, among them genistein, quercetin, naringin and *N-trans*-feruloyl tyramine were abundant in this sample. From the results described in section 3.5.1, quercetin and *N-trans*-feruloyl tyramine showed strong DPPH and lipase inhibitions, respectively. Thus, compared to other extracts, FWHS could have superior anti-lipase and antioxidant properties probably due to the presence of these two polyphenols within it. Moreover, to understand further the distinction in the samples based on their HPLC-quantified polyphenols, principal component analysis (PCA) was performed resulting in a two-dimensional map (Fig. 6 D). The first principal components account for 60.11 % of the total variance while the second components contribute 22.61 %. In the PCA plot, it was revealed that DHS and FDHS seemed similar in terms of the polyphenol profiles since they appeared close to each other on the left side of the PCA plot. However, WHS and FWHS displayed remarkable differences, demonstrated by their appearance on opposite sides of the PCA plot. Along the middle horizontal line which divides fermented and unfermented hempseed extracts, *N-trans-*feruloyl tyramine, genistein, naringin, gallic acid, apigenin and kaempferol were found to be important differentiating compounds in fermented products. Thus, the PCA scores plot presented a rapid and insightful classification of fermented and unfermented hempseed extracts using HPLC-quantified polyphenols.

## 4 Conclusion

The present investigation explored the potential of hempseed-rich polyphenols for promoting brain health, mitigating obesity, and preventing oxidative stress. All the tested hempseed samples exhibited no indications of cytotoxic effects on HepG2 cells, confirming their safety as potential phytomaterials. Fermented hempseed extracts displayed superior in vitro antioxidant, anti-obesity, and neuroprotective properties compared to their unfermented counterparts. These samples effectively inhibited ABTS and DPPH radicals and reduced reactive oxygen species (ROS) accumulation in HepG2 cells. Additionally, fermented hempseed demonstrated significant effects against acetylcholinesteras (AChE) activity, ROS accumulation, anti-fat potential, and anti-ageing ability by modulating the expression of related genes in *C. elegans*. Metabolomics analysis conducted on the extracts using HPLC and UHPLC-Q-TOF-MS/MS^2^ revealed an enhanced polyphenols profile after fermentation. Among these polyphenols, quercetin, gallic acid, and kaempferol showed excellent antioxidant potential, while *N-trans-*feruloyl tyramine displayed the highest anti-lipase potential. This study suggests that fermented hempseed, particularly fermented whole hempseed contains an elevated polyphenol profile, and could serve as antioxidant, anti-obesity and anti-neurodegeneration ingredients. However, there are two main limitations to the current study:

I. The analysis in this study focused solely on the phenolic profile of ethanol extracts from hempseeds. This means that the work only addressed a small fraction of the entire spectrum of bioactive compounds present in hempseeds. Future research should explore the health benefits of other compounds in hempseeds beyond ethanol-based phenolic extracts.
II. The conclusions regarding the health benefits of hempseeds were primarily drawn from in vitro cell line and *in vivo C. elegans* models, which have limitations, as it is difficult to predict if similar observations could be made in higher animals. Therefore, a more comprehensive conclusion on the health benefits of fermented hempseeds could be drawn from studies involving higher animals, such as mice. Thus, future studies will evaluate the inhibitory effects of fermented hempseed against obesity, oxidative stress, and neurodegenerative diseases in mouse models.

## CRediT authorship contribution statement

**Simon Okomo Aloo:** Writing – review & editing, Writing – original draft, Software, Methodology, Investigation, Formal analysis, Data curation, Conceptualization. **Kaliyan Barathikannan:** Writing – review & editing, Methodology, Investigation, Formal analysis. **Deog-Hwan Oh:** Writing – review & editing, Validation, Resources, Funding acquisition, Data curation, Conceptualization.

## Declaration of competing interest

The authors declare that they have no known competing financial interests or personal relationships that could have appeared to influence the work reported in this paper.

## Data Availability

No data was used for the research described in the article.
