# Supplementary material for: Polyphenol-rich fermented hempseed ethanol extracts improve obesity, oxidative stress, and neural health in high-glucose diet-induced Caenorhabditis elegans
Source: Food Chem X. 2024 Feb 20;21:101233. doi: 10.1016/j.fochx.2024.101233 (PMC10901904; doi:10.1016/j.fochx.2024.101233)
Supplement: Supplementary data 1 [file mmc1.pdf]

Polyphenol-rich fermented hempseed ethanol extracts improve obesity, oxidative stress, and neural health in high-glucose diet-induced *Caenorhabditis elegans*

Simon Okomo Aloo[1, 2], Kaliyan Barathikannan[1, 3], and Deog-Hwan Oh[1*]

[1]Department of Food Science and Biotechnology, College of Agriculture and Life Sciences, Kangwon National University, Chuncheon, Gangwon-do 24341, Republic of Korea

[2]Department of Food Science and Nutrition, Faculty of Agriculture and Food Security, Tom Mboya University, 199-40300, Kenya

[3]Agricultural and Life Science Research Institute, Kangwon National University, Chuncheon 24341, Republic of Korea

*Corresponding author: Deog-Hwan Oh

Email: deoghwa@kangwon.ac.kr

Supplementary

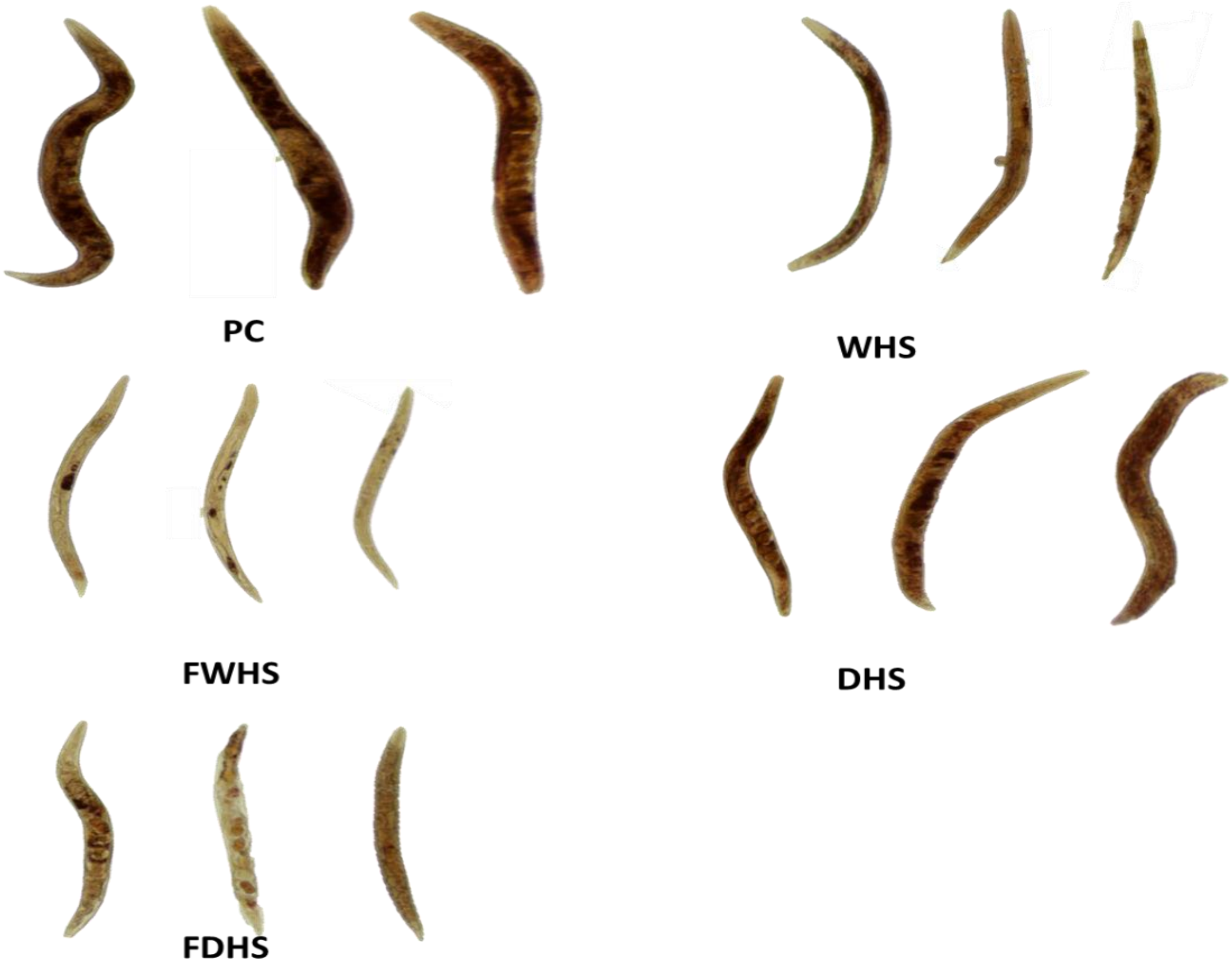

Figure 1. Oil red fluorescence images of *C. elegans* supplemented with glucose

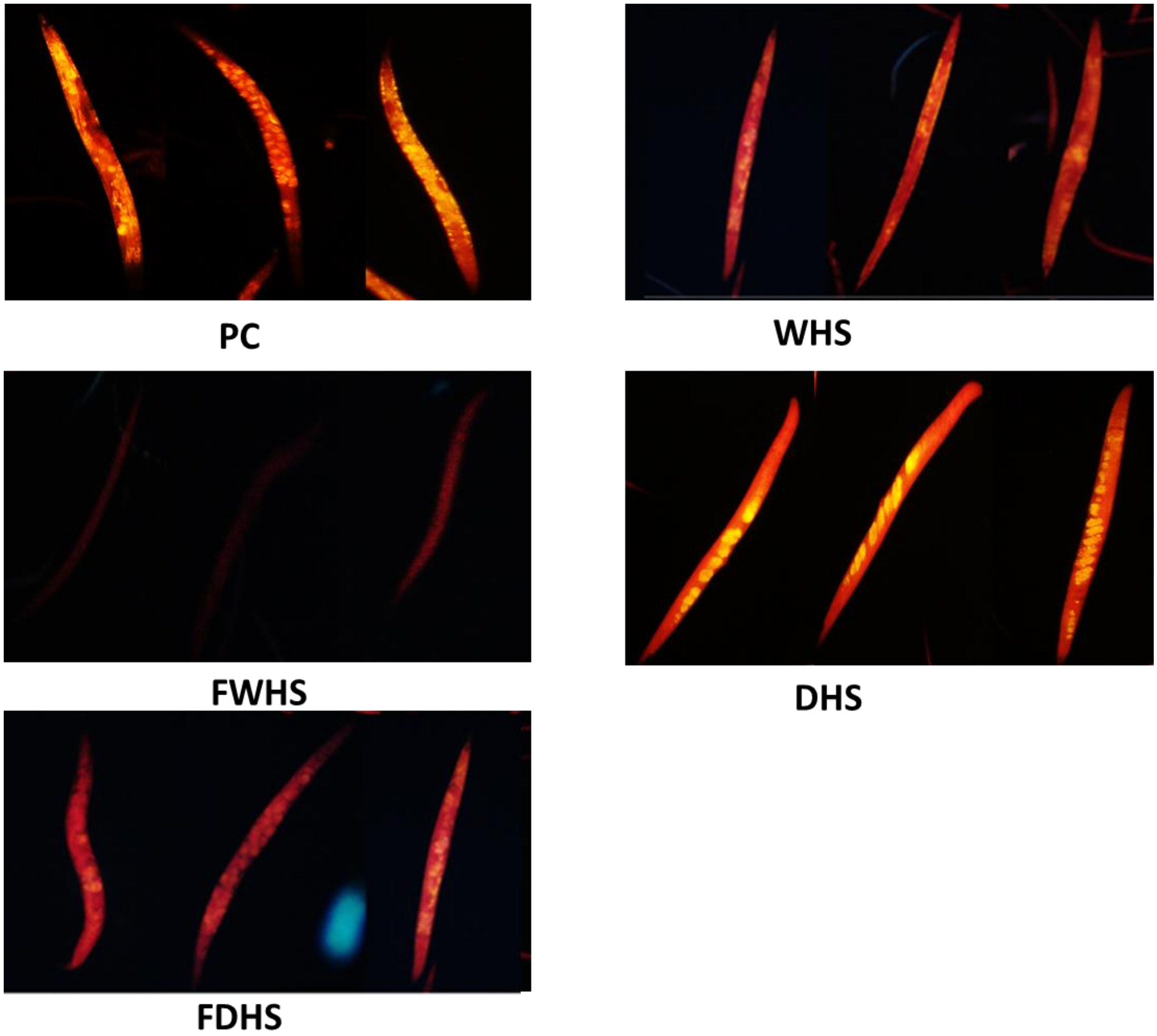

Figure 2. Oil red fluorescence images of *C. elegans* supplemented with glucose

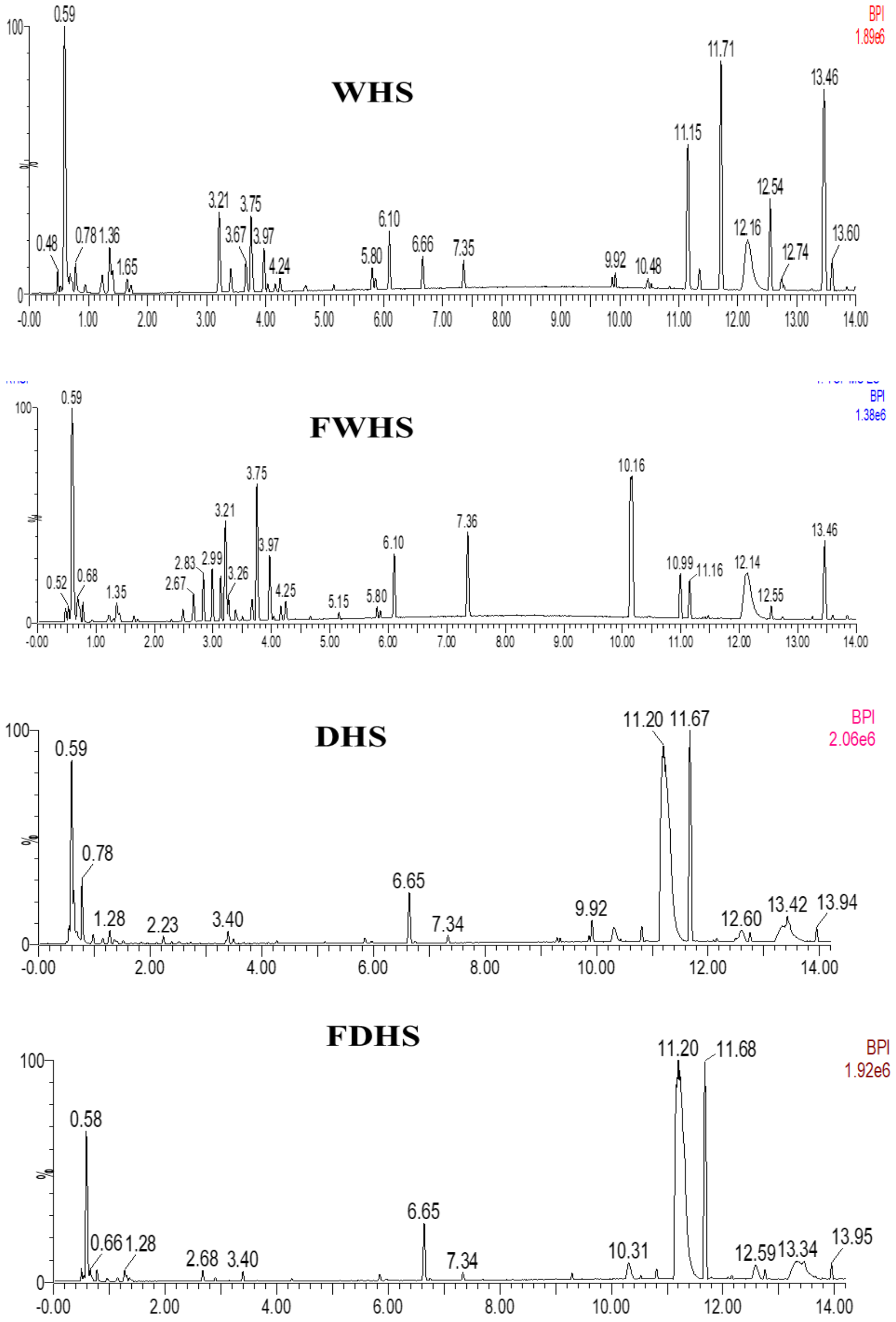

Supplementary figure 3. Obtained UHPLC-Q-TOF-MS/MS^2 chromatograms

Supplementary Table 1. *Caenorhabditis elegans* primers for detection of gene expression

Gene name	Forward primer	Reverse primer
Daf-16	CCAGACGGAAGGCTTAAACT	ATTCGCATGAAACGAGAATG
sod-1	ACGCTCGTCACGCTTTAC	TCTTCTGCCTTGTCTCCG
sod-2	GGCATCAACTGTCGCTGT	ACAAGTCCAGTTGTTGCC
Fat-6	CAACTTCCATCACACATTCCC	TCCTCGTTGAATATCACATCC
Fat-7	TTTCCACCACACATTCCCAC	TCTTCACTTCCGTGATTGGC
β-actin 2	ATCGTCCTCGACTCTGGAGAT	TCACGTCCAGCCAAGTCAAG
Ace-1	AGTGGGCTCCTGTTCGAGAA	CCAATAGAAAATCACCATCGACAA
Ace-2	CAATAATCAACTCATGGGCATCA	TTTTCGCGAGACGAAACGA

Supplementary Table 2. Effects of fermentation on antioxidant capacity of hempseed ethanol extracts

	DPPH (%)				ABTS (%)			
	**50 μg/mL**	**100 μg/mL**	**200 μg/Ml**	**400 μg/mL**	**50 μg/mL**	**100 μg/mL**	**200 μg/mL**	**400 μg/mL**
WHS	1.00 ± 0.44	3.96 ± 0.24	6.36 ± 1.01	7.30 ± 1.30	0.11 ± 0.11	2.37 ± 0.20	5.64 ± 0.39	14.00 ± 0.51
FWHS	7.39 ± 4.09	11.96 ± 5.24	20.68 ± .79	29.51 ± 7.26	11.11 ± 0.44	24.26 ± 0.18	48.22 ±0.20	78.27 ± 0.63
DHS	3.26 ± 1.89	3.32 ± 1.16	4.71 ± 1.11	5.98 ± 0.79	0.59 ± 0.62	1.00 ± 0.51	2.24 ± 0.20	2.10 ± 0.51
FDHS	3.95 ± 2.12	6.52 ± 1.13	11.99 ± 2.41	17.99 ± 1.91	3.46 ± 0.30	7.90 ± 0.58	16.24 ±0.17	30.18 ± 0.83
BHA	26.41±3.09	45.63 ± 2.65	60.34 ± 0.72	86.25 ± 1.94	81.51 ± 0.58	96.94 ± 0.16	≥99.99	99.99
AA	81.81±2.16	91.88 ± 0.46	95.67 ± 0.05	≥99.99	95.14 ± 0.04	≥99.99	≥99.99	99.99

Absorbance measurement: absorbance recorded at 700nm. 1) The positive controls employed were butylated hydroxyanisole (BHA) and ascorbic acid (AA), respectively. Different hempseed samples included WHS (whole hempseed), FWHS (fermented whole hempseed), DHS (dehulled hempseed), and FDHS (fermented dehulled hempseed).

Supplementary Table 3. Correlation between polyphenols and antioxidant and lipase inhibition capacity

**Polyphenol**	**Parameter**	**Pearson's r**	**p**
TPC	ABTS	0.988	0.012
	DPPH	0.936	0.064
	Lipase	0.959	0.041
Quercetin	ABTS	0.585	0.415
	DPPH	0.502	0.498
	Lipase	0.761	0.239
N-trans-feruloyltyramine	ABTS	0.660	0.340
	DPPH	0.510	0.490
	Lipase	0.779	0.221
Apigenin	ABTS	0.891	0.109
	DPPH	0.777	0.223
	Lipase	0.880	0.120
Rutin	ABTS	-0.341	0.659
	DPPH	-0.480	0.520
	Lipase	-0.134	0.866
p- coumaric acid	ABTS	-0.018	0.982
	DPPH	-0.131	0.869
	Lipase	-0.167	0.833
Gallic acid	ABTS	0.939	0.061
	DPPH	0.871	0.129
	Lipase	0.869	0.131
	ABTS	0.939	0.061
Naringin	DPPH	0.871	0.129
	Lipase	0.869	0.131
Ferulic acid	ABTS	-0.787	0.213
	DPPH	-0.642	0.358
	Lipase	-0.838	0.162
Kaempferol	ABTS	0.524	0.476
	DPPH	0.677	0.323
	Lipase	0.370	0.630
	Lipase	0.880	0.120
Genistein	ABTS	0.256	0.744
	DPPH	0.072	0.928
	Lipase	0.421	0.579
Catechin	ABTS	-0.545	0.455
	DPPH	-0.375	0.625
	Lipase	-0.674	0.326
Caffeic acid	ABTS	-0.474	0.526
	DPPH	-0.283	0.717
	Lipase	-0.577	0.423
